# Assessment of Fluid Status by Bioimpedance Analysis and Central Venous Pressure Measurement and Their Association with the Outcomes of Severe Acute Kidney Injury

**DOI:** 10.3390/medicina57060518

**Published:** 2021-05-22

**Authors:** Justina Karpavičiūtė, Inga Skarupskienė, Vilma Balčiuvienė, Rūta Vaičiūnienė, Edita Žiginskienė, Inga Arūnė Bumblytė

**Affiliations:** 1 Department of Nephrology, Medical Academy, Lithuanian University of Health Sciences, Eivenių 2, LT-50161 Kaunas, Lithuania; i.skarupskiene@gmail.com (I.S.); ruta.vaiciuniene@kaunoklinikos.lt (R.V.); Edita.Ziginskiene@kaunoklinikos.lt (E.Ž.); Inga.Bumblyte@kaunoklinikos.lt (I.A.B.); 2 Hospital of Lithuanian University of Health Sciences, Eivenių 2, LT-50161 Kaunas, Lithuania; vilma.balciuviene@kaunoklinikos.lt

**Keywords:** hyperhydration, hypervolemia, bioimpedance, acute kidney injury, outcomes of AKI, hemodialysis

## Abstract

*Background and Objectives:* Fluid disbalance is associated with adverse outcomes in critically ill patients with acute kidney injury (AKI). In this study, we intended to assess fluid status using bioimpedance analysis (BIA) and central venous pressure (CVP) measurement and to evaluate the association between hyperhydration and hypervolemia with the outcomes of severe AKI. *Materials and Methods:* A prospective study was conducted in the Hospital of the Lithuanian University of Health Sciences Kauno Klinikos. Forty-seven patients treated at the Intensive Care Unit (ICU) with severe AKI and a need for renal replacement therapy (RRT) were examined. The hydration level was evaluated according to the ratio of extracellular water to total body water (ECW/TBW) of bioimpedance analysis and volemia was measured according to CVP. All of the patients were tested before the first hemodialysis (HD) procedure. Hyperhydration was defined as ECW/TBW > 0.39 and hypervolemia as CVP > 12 cm H_2_O. *Results:* According to bioimpedance analysis, 72.3% (*n* = 34) of patients were hyperhydrated. According to CVP, only 51.1% (*n* = 24) of the patients were hypervolemic. Interestingly, 69.6% of hypovolemic/normovolemic patients were also hyperhydrated. Of all study patients, 57.4% (*n* = 27) died, in 29.8% (*n* = 14) the kidney function improved, and in 12.8% (*n* = 6) the demand for RRT remained after in-patient treatment. A tendency of higher mortality in hyperhydrated patients was observed, but no association between hypervolemia and outcomes of severe AKI was established. *Conclusions:* Three-fourths of the patients with severe AKI were hyperhydrated based on bioimpedance analysis. However, according to CVP, only half of these patients were hypervolemic. A tendency of higher mortality in hyperhydrated patients was observed.

## 1. Introduction

Early and adequate fluid restoration is fundamental to increase intravascular volume and maintain organ perfusion in the treatment of critically ill patients [1]. However, there is growing evidence that aggressive fluid therapy can lead to fluid overload and further organ damage [2]. Fluid accumulation occurs not only as a consequence of fluid therapy but also in sepsis due to the relaxation of complement factors, cytokines, prostaglandin products, and altered organ microcirculation [3]. Excess fluid can cause visceral oedema and intra-abdominal hypertension, which result in decreased renal perfusion and glomerular filtration [1,4]. Several observational studies have linked a positive fluid balance, especially before the onset of renal replacement therapy, to the occurrence of acute kidney injury (AKI) and mortality [5,6,7,8].

Several methods exist to evaluate fluid status: physical examination, chest radiography, chest ultrasound, central venous pressure (CVP), and bioimpedance analysis (BIA). CVP measurement is the most commonly used manner to guide fluid restoration, but it is often noted that it poorly indicates volume responsiveness [9,10]. BIA is widely used in the assessment of the hydration status of chronical kidney disease (CKD) and dialyzed patients. The clinical benefit of bioimpedance is the evaluation of total extracellular fluid overload, rather than only intravascular fluid. Few data exist on the use of bioimpedance for the evaluation of the hydration status of the patients with AKI. 

The aim of our study was to establish the frequency of hypervolemia and hyperhydration detected by CVP and bioimpedance analysis and to evaluate how hydration condition influences the outcomes of severe AKI. 

## 2. Materials and Methods

This prospective study included 47 consecutive patients ill with 3rd stage of AKI (according to KDIGO) and treated at the Intensive Care Unit (ICU) of the Hospital of the Lithuanian University of Health Sciences Kauno Klinikos (HLUHS KK) during the study period. All study patients were older than 18 years and had been ill with severe AKI requiring renal replacement therapy (RRT). The exclusion criteria were: established end-stage kidney disease with demand for RRT, pregnancy, large metal implants present in the body, limb amputation and temporary or constant cardiac stimulator. The study was approved by the Kaunas Regional Biomedical Research Ethics Committee (1 October 2013 No. BE-2-45) and State Data Protection Inspectorate (No. 2R-6201 (2.6-1.).

Bioimpedance analysis was performed and CVP was measured before the start of the first hemodialysis (HD). The hydration status was evaluated according to the ratio of extracellular water (ECW) and total body water (TBW), calculated by InBody S10, Biospace Co., Ltd. analyzer. The normal range for ECW/TBW was considered 0.36–0.39, as noted in the results sheet of the analyzer. According to this ratio, the patients were divided into two groups: (1) ECW/TBW ≤ 0.39 (hypohydrated/normohydrated), and (2) ECW/TBW > 0.39 (hyperhydrated). The patients were also divided into two groups according to CVP: (1) CVP ≤ 12 cmH_2_O (hypovolemic/normovolemic), and (2) CVP > 12 cmH_2_O (hypervolemic). The balance of daily fluids was calculated by deducting the amount of the fluid lost during the day from the amount of fluid received during the day. The clinical signs of hydration status were not analyzed. The BIA parameters of the investigated patients in the groups of survivors and the nonsurvivors were analyzed. In addition, relationships between the groups of hypovolemia/normovolemia, hypervolemia (according to CVP), hypohydration/normohydration, hyperhydration (according to ECW/TBW) were investigated. 

Variables were expressed as frequencies, percentages for discrete factors, and mean values ± standard deviation (SD) for continuous factors. Statistical analysis was performed using SPSS 22.0 (Statistical Package for Social Science 22 for Windows). For the comparison of two independent groups’ quantitative values, the Student’s *t*-test was applied. Accordingly, for non-normally distributed data, non-parametric methods—Mann–Whitney U and Kruskal–Wallis tests—were employed. For the analysis of the qualitative data, the Chi-square (χ^2^) test of compatibility and independence was applied. Taking into consideration the distribution of the variables, we applied Pearson’s or Spearman’s correlation coefficient. A *p*-value <0.05 was considered to be statistically significant.

## 3. Results

A total of 47 severe AKI patients treated in the ICU of HLUHS KK were involved in the study. The demographic and clinical data of the investigated patients are presented in Table 1.

The mean age of the patients was 70.13 ± 13.7 years. Three-fourths of the patients were older than 62. For 14.9% (*n* = 7) of patients, only kidney failure was detected. They were admitted to ICU for post-resuscitation care and required additional monitoring because of life-threatening hyperkalemia. For 80.9% (*n* = 38) of patients, concurrently lungs were affected, for 23.4% (*n* = 11) liver was affected, for 55.3% (*n* = 26) heart was affected. Sepsis with a septic shock was established for nearly half of these patients; for 72.3%, artificial pulmonary ventilation (APV) was applied, and for 85.1%, multiorgan failure (MOF) was established.

We measured CVP and performed BIA before the start of the first hemodialysis procedure for AKI patients: 51.1% (*n* = 24) of the patients were hypervolemic (CVP was >12 cmH_2_O), and 72.3% (*n* = 34) of patients were hyperhydrated (ECW/TBW was >0.39). The number of hyperhydrated patients before the first RRT procedure was statistically significantly higher than those who were hypohydrated/normohydrated (*p* = 0.002).

**Figure 1:**
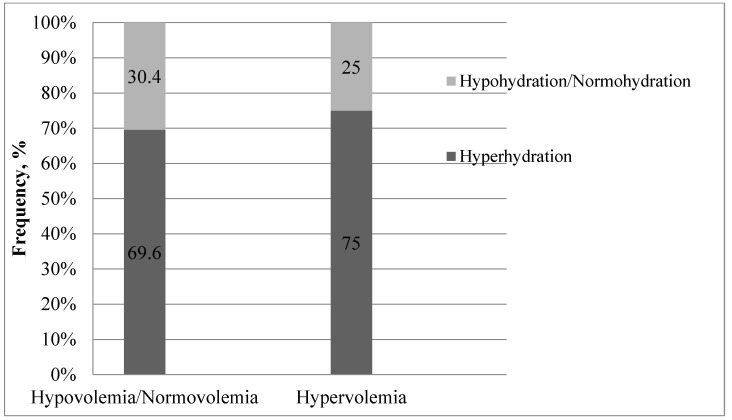
Frequency of distribution of hydration groups in different volemia groups according to central venous pressure (CVP) before the first procedure of renal replacement therapy.

We found that a major portion of the hypervolemic patients were statistically significantly more often hyperhydrated than hypohydrated/normohydrated patients (*p* = 0.014). Interestingly, 69.6% of hypovolemic/normovolemic investigated patients were also hyperhydrated (Figure 1).

We found that, in hypervolemic patients with severe AKI, ICW/body weight, ECW/body weight, and TBW/body weight measured by BIA were statistically significantly lower than in hypovolemic/normovolemic patients. This shows a larger quantity of fluid of these patients in the major blood vessels, but not in the body tissues (Table 2).

We established that 57.4% (*n* = 27) of the investigated patients died, the kidney function of 29.8% (*n* = 14) improved, and for 12.8% (*n* = 6) the demand for RRT remained after in-patient treatment. We did not find a relationship between hypervolemia according to CVP and mortality in severe AKI patients (Table 3).

Only the tendency of higher mortality was observed in hyperhydrated patients according to BIA (Table 4).

**Figure 2:**
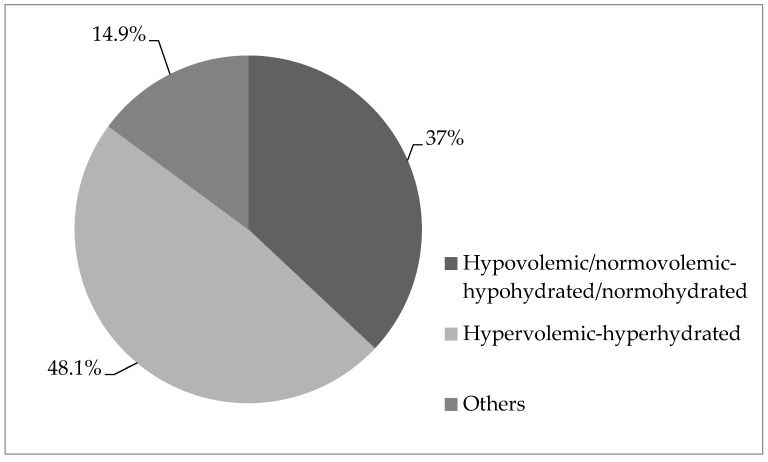
The percentage distribution of the patients according to volemia and hydration in the group of nonsurvivors.

We analyzed the distribution of volemia and hydration in non-survivors before the first RRT procedure: 48.1% of patients were hypervolemic-hyperhydrated, and 37% were hypovolemic-hypohydrated (Figure 2). 

Thus, neither hypervolemia according to CVP nor hyperhydration established by the bioimpedance method significantly influenced mortality. The same result was confirmed using continuous variables. We did not find the cutoff value of ECW/TBW or CVP for mortality. 

## 4. Discussion

Our study indicates that fluid overload may be diagnosed more precisely when tested using several methods. CVP is influenced by many factors such as blood pressure, heart rate, cardiac output, errors related to positioning of the zero level or reading errors, thus it is difficult to determine fluid balance based on a single CVP value [10]. In our research, hypervolemic patients accounted for about half of all subjects, as many as 72.3% of patients had an ECW/TBW > 0.39 and were hyperhydrated, and nearly half of the patients were both hypervolemic and hyperhydrated. A large proportion of subjects had sepsis, in which inflammatory cytokines increase capillary permeability, which may have led to hyperhydration. We found that a major portion of the patients in our study were both hypervolemic and hyperhydrated, and mortality was higher in this group. Unfortunately, we did not find a significant association between hypervolemia/hyperhydration and patient outcome. We believe that statistically significant results were not obtained due to the small sample size of our study. Thus, a higher number of patients is needed to evaluate the influence of fluid metabolism disorders for the outcomes of severe AKI patients.

Several studies on the hydration status of patients with AKI requiring RRT have been conducted. One study in which hydration was assessed using the same method as in our study was conducted by K.H. Park and colleagues. This prospective observational study included 31 patients who were admitted to the ICU and required continuous renal replacement therapy (CRRT). Cox regression analysis revealed a statistically insignificant association between ECW/TBW ≥ 0.41 and 28 day mortality, but the area under the curve of ECW/TBW was 0.73 (95% confidence interval, 0.54 to 0.92), which was statistically significant (*p* = 0.037). Thus, it was concluded that hydration status could be evaluated by bioimpedance in critically ill patients requiring CRRT and it has prognostic value for treatment outcomes [11]. 

D. Slobod and co-authors evaluated the hydration of ICU patients according to ECW/TBW ratio and investigated the effect of volume status on the duration of mechanical ventilation, 28 day mortality and AKI requiring RRT. No association between ECW/TBW ratio at day 1 and day 28 was found, but it was revealed that bioimpedance-measured ECW/TBW ratio on day 1 of admission to the ICU was associated with time on the ventilator (*p* = 0.05) [12]. In our study, we also found that higher proportion of hyperhydrated patients suffered from MOF and were on APV compared to hypohydrated/normohydrated patients.

Statistical vector analysis of BIA appears to be a feasible method to evaluate the fluid status of critically ill patients [13]. A.C. Hise et al. used this approach to assess the hydration status of 224 critically ill patients under intensive care at the time of AKI diagnosis and observed a statistically significant difference in hydration between survivors and nonsurvivors [14]. Di Somma et al. found that using vector analysis of BIA has significant prognostic value for patients with acute heart failure in brain natriuretic peptide (BNP) “grey values” (100–400 pg/mL) and could help to reduce subsequent cardiovascular events [15]. Some studies identified hyperhydration in severe patients as an independent risk factor for hospital death and found a significant association between hyperhydration and mortality [13,16,17,18,19,20]. In our study, only a tendency of higher mortality was found in patients with hypervolemia–hyperhydration, but it was not statistically significant, probably due to the small sample size.

Other studies evaluated the association of positive fluid balance or fluid overload (weight gain >5–10% from baseline) with outcomes of patients with AKI. For example, J.S. Kim and co-authors analyzed the data of 341 patients treated in ICU with AKI. Survival was significantly lower in patients with fluid overload (at least 10% weight gain from baseline) 3 days before the start of RRT compared to patients without fluid overload (*p* < 0.001). However, among patients without sepsis or a low sequential organ failure assessment (SOFA) score, no statistically significant effect on survival was observed, regardless of the presence of fluid overload [6].

C.W. Woodward and colleagues reviewed the data of 481 patients requiring RRT for AKI and analyzed the association between fluid overload and major adverse renal events (defined as mortality, dependence on RRT, and inability to recover 50% of baseline eGFR (if dependence on HD regressed) for up to 90 days after discharge). Patients with an excess of fluid ≤10% were less likely to experience major adverse kidney events than those with fluid overload greater than 10% (71.6% vs. 79.4%; *p* = 0.047). Fluid overload ≥10% was also found to be independently associated with 82% increased odds of hospital mortality (*p* = 0.004) and 2.5 fewer ventilator-free days (*p* = 0.044). Thus, fluid overload was identified as a potentially modifiable risk factor that should be further examined in interventional studies [19]. G.E. Hatton and co-authors found that positive fluid balance (>2 L) is detected in half of severely injured patients within 48 h after trauma, and fluid overload is independently and gradually related to the development of AKI [20]. 

D.K. Li and colleagues performed a retrospective analysis of more than 9000 ICU patients and evaluated the association between hypervolemia according to the mean CVP level and 28 day mortality. It was found that elevated CVP level correlates with poor outcomes and prolonged treatment [21]. However, a study by H. Uthoff et al. showed that patients with acute heart failure and CVP  < 10 cm H_2_O were more likely to develop worsening renal function during the first 24 h of hospitalization compared to patients with CVP > 15 cm H_2_O [22]. This implies that the evaluation of volume status is complex and should be individualized.

Our prospective study is one of the few attempts to evaluate the hydration of patients with AKI using the bioimpedance method, in addition to such common parameters as volemia, diuresis, and fluid balance. It is well known that tissue hydration depends on many factors: volemia, cardiac function, oncotic pressure in blood vessels, the effect of cytokines on cell membranes (in the case of sepsis), etc. Interestingly, data of our study showed that almost 70% of hypovolemic/normovolemic patients were hyperhydrated. This could be explained by the fact that more than half of our patients were diagnosed with heart failure, a quarter with liver damage, and about 60% of patients had sepsis, which could cause fluid to enter the tissues. The limitation of our study is the choice of a cohort of severely ill patients in which hyperhydration was still common, although not correlated with hypervolemia. Thus, to address the remaining questions, more detailed studies should be performed including patients with a milder condition and including more parameters (albumin, cardiac ultrasound, etc.).

## 5. Conclusions

Three-fourths of the patients with severe AKI were hyperhydrated based on bioimpedance analysis. However, according to CVP, only half of these patients were hypervolemic. It shows that fluid overload may be diagnosed more precisely when tested using several methods. A tendency of higher mortality in hyperhydrated patients was observed.

## Figures and Tables

**Table 1:** The demographic and clinical data of the investigated patients (*n* = 47).

Characteristic of Patients	Value
Gender: Male/female, *n* (%)	24 (51.1)/23 (48.9)
Age (mean ± SD), years	70.13 ± 13.7
Length of stay (mean ± SD) in ICU, days	26.04 ± 28.3
Length of stay till getting into ICU (mean ± SD), days	4.45 ± 12.9
Length of stay in ICU till the beginning of RRT (mean ± SD), days	1.32 ± 2.0
Chronic kidney disease (the 1st CKD stage), *n* (%)	2 (4.3)
Chronic kidney disease (the 2nd–4th CKD stage), *n* (%)	15 (31.9)
Use of vasopressors, *n* (%)	25 (53.2)
Application of APV, *n* (%)	34 (72.3)
Heart failure, *n* (%)	26 (55.3)
Liver damage, *n* (%)	11 (23.4)
Sepsis without a septic shock, *n* (%)	7 (14.9)
Sepsis with a septic shock, *n* (%)	21 (44.7)
Before the first RRT procedure:	
Pulmonary oedema *n* (%)	8 (17)
Oliguria, *n* (%)	39 (83)
Serum potassium (mean ± SD), mmol/L	5.20 ± 1.1
Serum sodium (mean ± SD), mmol/L	139.32 ± 8.9
Serum urea (mean ± SD), mmol/L	32.64 ± 11.8
Serum creatinine (mean ± SD), µmol/L	511.83 ± 169.3
pH of blood (mean ± SD)	7.28 ± 0.1
Serum bicarbonate (HCO_3_) (mean ± SD), mmol/L	16.3 ± 3.9

ICU—intensive care unit, RRT—renal replacement therapy, APV—artificial pulmonary ventilation, BP—arterial blood pressure, CKD—chronic kidney disease.

**Table 2:** The comparison of bioimpedance parameters before the first procedure of the renal replacement therapy in separate CVP groups.

BIA Parameters	CVP ≤ 12 cm H_2_O (*n* = 23)	CVP > 12 cm H_2_O (*n* = 24)	*p*-Value
ICW/body weight	0.36 ± 0.07	0.31 ± 0.06	0.013
ECW/body weight	0.26 ± 0.06	0.23 ± 0.04	0.042
TBW/body weight	0.63 ± 0.13	0.55 ± 0.10	0.02
ECW/TBW	0.41 ± 0.11	0.42 ± 0.04	0.317

CVP—central venous pressure, ICW—intracellular water, ECW—extracellular water, TBW—total body water.

**Table 3:** The outcomes of the patients in different groups according to CVP.

Outcomes	CVP before the First RRT Procedure		
	≤12 cm H_2_O (*n* = 23)	>12 cm H_2_O (*n* = 24)	*p*-Value
Survivors, *n* (%)	9 (39.1)	11 (45.8)	0.642
Nonsurvivors, *n* (%)	14 (60.9)	13 (54.2)	0.642

**Table 4:** The outcomes of the patients in different extracellular water to total body water (ECW/TBW) groups.

Outcomes	ECW/TBW before the First RRT Procedure		
	≤0.39 (*n* = 13)	>0.39 (*n* = 34)	*p*-Value
Survivors, *n* (%)	8 (61.5)	12 (35.3)	0.104
Nonsurvivors, *n* (%)	5 (38.5)	22 (64.7)	0.104

## Data Availability

Not applicable.
